# Phospholipid Species in Newborn and 4 Month Old Infants after Consumption of Different Formulas or Breast Milk

**DOI:** 10.1371/journal.pone.0162040

**Published:** 2016-08-29

**Authors:** Olaf Uhl, Manja Fleddermann, Christian Hellmuth, Hans Demmelmair, Berthold Koletzko

**Affiliations:** Ludwig-Maximilians-University of Munich, Dr. von Hauner Children’s Hospital, Medical Centre of LMU Munich, München, Germany; Universidade de Sao Paulo Instituto de Quimica, BRAZIL

## Abstract

**Introduction:**

Arachidonic acid (AA) and docosahexaenoic acid (DHA) are important long-chain polyunsaturated fatty acids for neuronal and cognitive development and are ingredients of infant formulae that are recommended but there is no evidence based minimal supplementation level available. The aim of this analysis was to investigate the effect of supplemented AA and DHA on phospholipid metabolism.

**Methods:**

Plasma samples of a randomized, double-blind infant feeding trial were used for the analyses of phospholipid species by flow-injection mass spectrometry. Healthy term infants consumed isoenergetic formulae (intervention formula with equal amounts of AA and DHA—IF, control formula without additional AA and DHA—CF) from the first month of life until the age of 120 days. A group of breast milk (BM) -fed infants was followed as a reference.

**Results:**

The plasma profile detected in newborns was different from 4 month old infants, irrespective of study group. Most relevant changes were seen in higher level of LPC16:1, LPC20:4, PC32:1, PC34:1 and PC36:4 and lower level of LPC18:0, LPC18:2, PC32:2, PC36:2 and several ether-linked phosphatidylcholines in newborns. The sum of all AA and DHA species at 4 month old infants in the CF group showed level of 40% (AA) and 51% (DHA) of newborns. The supplemented amount of DHA resulted in phospholipid level comparable to BM infants, but AA phospholipids were lower than in BM infants. Interestingly, relative contribution of DHA was higher in ether-linked phosphatidylcholines in CF fed infants, but IF and BM fed infants showed higher overall ether-linked phosphatidylcholines levels.

**Conclusion:**

In conclusion, we have shown that infant plasma phospholipid profile changes remarkably from newborn over time and is dependent on the dietary fatty acid composition. A supplementation of an infant formula with AA and DHA resulted in increased related phospholipid species.

## Introduction

After birth, breast milk consumption is assumed to be the best nutrition to support optimal infantile development. Thus, infant formulae should be designed according to human milk composition to meet infant’s needs. Besides protein and carbohydrate content, lipid composition is essential, e.g. an essential fatty acid ratio of linoleic acid to alpha-linolenic acid ratio in the range of 5–15 is recommended [1, 2]. Arachidonic acid (AA) and docosahexaenoic acid (DHA) are the most important essential long-chain polyunsaturated fatty acids (LC-PUFA) which are recommended ingredients for infant formula, but there are no evidence based minimal supplementation level available. A series of studies have shown that LC-PUFA supplementation of infant formula improved the fatty acids status in different matrices such as plasma and red blood cells in infants [3]. Phospholipids are membrane lipids and known to be a good biomarker for LC-PUFA status, due to a high content of these essential fatty acids [4]. Thus, the analysis of plasma phospholipids is used to assess the bioavailability of supplemented LC-PUFA. For the development of new infant formulae, fatty acid analyses of plasma or red blood cell phospholipids are usually used to determine the fatty acid status [5].

Phospholipids are molecules with a lipophilic fatty acid side chain and a hydrophilic phosphate containing head group. Hundreds of diverse molecular species of phospholipids that differ in kind of head group, chain length, number and positions of double bonds and chemical bindings of fatty acids to the glycerol or sphingosine backbone [6] are known. There are several lipid molecules that contain fatty acid chains like AA or DHA. Thus, it seems to be important to know how supplemented LC-PUFA is incorporated in the lipidome and which metabolites reflect LC-PUFA best in infant plasma.

To investigate this question, we chose a previously published randomized, double-blind intervention trial which investigated a new designed low protein intervention formula (IF) supplemented with equal amounts of AA and DHA (7.2 mg/ 100 mL) in comparison to a control formula (CF) without AA and DHA [7]. Fully breastfed infants were followed as a non-randomized reference group. The supplemented AA and DHA in the IF resulted in higher percentages of these fatty acids in plasma glycerophospholipids in comparison to CF [7]. The aim of this analysis was to investigate the effect of supplemented AA and DHA and the change of different plasma phospholipid species in the first month of life. We focused our analysis on choline containing phospholipid species, namely lyso-phosphatidylcholines (LPC), diacyl-phosphatidylcholines (PC), ether-linked phosphatidylcholines (PCe) and sphingomyelines (SM), which covered 95% of phospholipids [8].

## Methods

### Study

We analysed plasma samples of infants from the BeMIM study (Belgrade-Munich Infant Milk trial), a randomized, double-blind, controlled intervention study. Details on the study design, participating subjects, as well as details about infant formula composition have been previously published [7]. Briefly, healthy term infants were recruited until the age of 28 days and randomised to receive either an intervention formula (IF) with AA and DHA (from egg and fish oils) or a control formula (CF) without AA and DHA. Furthermore, IF had a lower protein content (1.89 g/100 kcal) and a higher fat content (5.3 g/ 100 kcal), compared to the CF (2.2 g protein and 4.9 g fat per 100 kcal). IF was enriched in alpha-lactalbumin and supplemented with free L-phenylalanine and L-tryptophan to meet required contents [2]. A reference group of exclusively breastfed infants was included (BM). Plasma samples were taken at 120 days of age and from a subgroup of subjects plasma samples were taken at recruitment before the study started (newborn, 0–5 days after birth). Newborn and subjects did not fully coincide after the intervention due to drop outs and thus a longitudinal analysis was unrewarding. The study was approved by the Clinical Center Serbia Ethical Committee. Written informed consent was obtained from all participating families. Analyses of this publication have been performed as secondary analyses of a randomized controlled trial and got approval from the Ethical Committee of Ludwig-Maximilians University of Munich, Germany. The original study was registered at Clinical Trials.gov (NCT01094080). The fatty acid compositions of both infant formulas are shown in Table 1.

**Table 1:** Fatty acid content (g/100 mL) of the studied infant formulas.

	Intervention Formula	Control Formula
C12:0	0.2	0
C14:0	0.1	0
C16:0	0.8	0.9
C16:1	0	0
C18:0	0.1	0.1
C18:1	1.5	1.4
C18:2	0.7	0.7
C18:3	0.1	0.1
C20:4 (mg/100 mL)	7.2	0
C22:6 (mg/100 mL)	7.2	0

### Analysis of glycerophospholipids

The plasma samples were analysed as described in S1 File and in previous studies [9–11]. Briefly, 10 μl plasma were diluted with methanol, containing internal standards, to correct for any effects appearing during sample preparation and ionization. After centrifugation, supernatants were used for flow-injection mass spectrometry analysis. The liquid chromatographic system (Agilent, Waldbronn, Germany) was coupled to a triple quadrupole mass spectrometer (QTRAP4000, Sciex, Darmstadt, Germany) with an electrospray ionization source. Mass spectrometric analysis was run in Multiple Reaction Monitoring mode. Quantification of metabolites has been done by comparison of signal-to-internal standard-ratios between samples and commercial available lyophilized aliquots of control plasma (Recipe, Germany) as one point calibration. The entire analytical process was post-processed by Analyst 1.5.1 and the isotopomer correction for up to M+4 was applied by R (programming language, version 3.0.1).

The full analytical analysis comprised LPC, PC, PCe, SM, acylcarnitines and sum of hexoses, but data analyses focus only on phospholipids. As a point to note, the analytical technique applied here is not capable of determining the position of the double bonds and the distribution of carbon atoms between fatty acid side chains. The polar lipids are mentioned as X:Y. In this nomenclature, X is the length of the carbon chain, Y is the number of double bonds. There will be no differentiation between ether-linked and vinyl ether-linked phosphatidylcholines. However in PC, the ether-linked species are dominating and thus the interpretation of fatty acids follows this assumption. Furthermore, it needs to be addressed that there might be more than one species covered by one mass transition and the most concentrated species (according to lipidmaps.org [12] and human metabolome data base [13]) was used as species name.

### Data analysis

A number of 484 plasma samples were measured randomly in batches composed of 81 samples, 6 quality control samples and 9 standards. Aliquots of a pooled plasma sample were used as quality control samples for intra- and inter-batch variation. Coefficients of variation were used as the criteria of quality. Metabolites are presented as median and interquartile ranges (IQR) in μmol/L. A principal component analyses (PCA) was carried out to identify possible separation of subjects according to grouping. Mann-Whitney-U tests were performed to investigate differences between groups. Significance was accepted at p <0.05. Due to multiple testing, the level of significance for p-values was corrected according to Bonferroni to p-values <5.49E-04. All statistical tests were performed with SPSS software version 21 (IBM, NY, USA) or Excel 2010.

## Results

A total of 484 plasma samples (231 newborn, 79 CF, 83 IF and 91 BM) of the BeMIM trial have been determined for 425 phospholipids. Eleven LPC, 26 PC, 27 PCe and 27 SM were determined with intra-batch and inter-batch coefficient of variation less than 30% and considered for further statistical analysis. Details on the anthropometric data have been previously published [7]. Briefly, the mean age of the 231 newborn was 3 days (range 2 to 5) with a mean birthweight of 3437 g (SD 373 g).

### Changes from birth to 4 month of age

**Fig 1:**
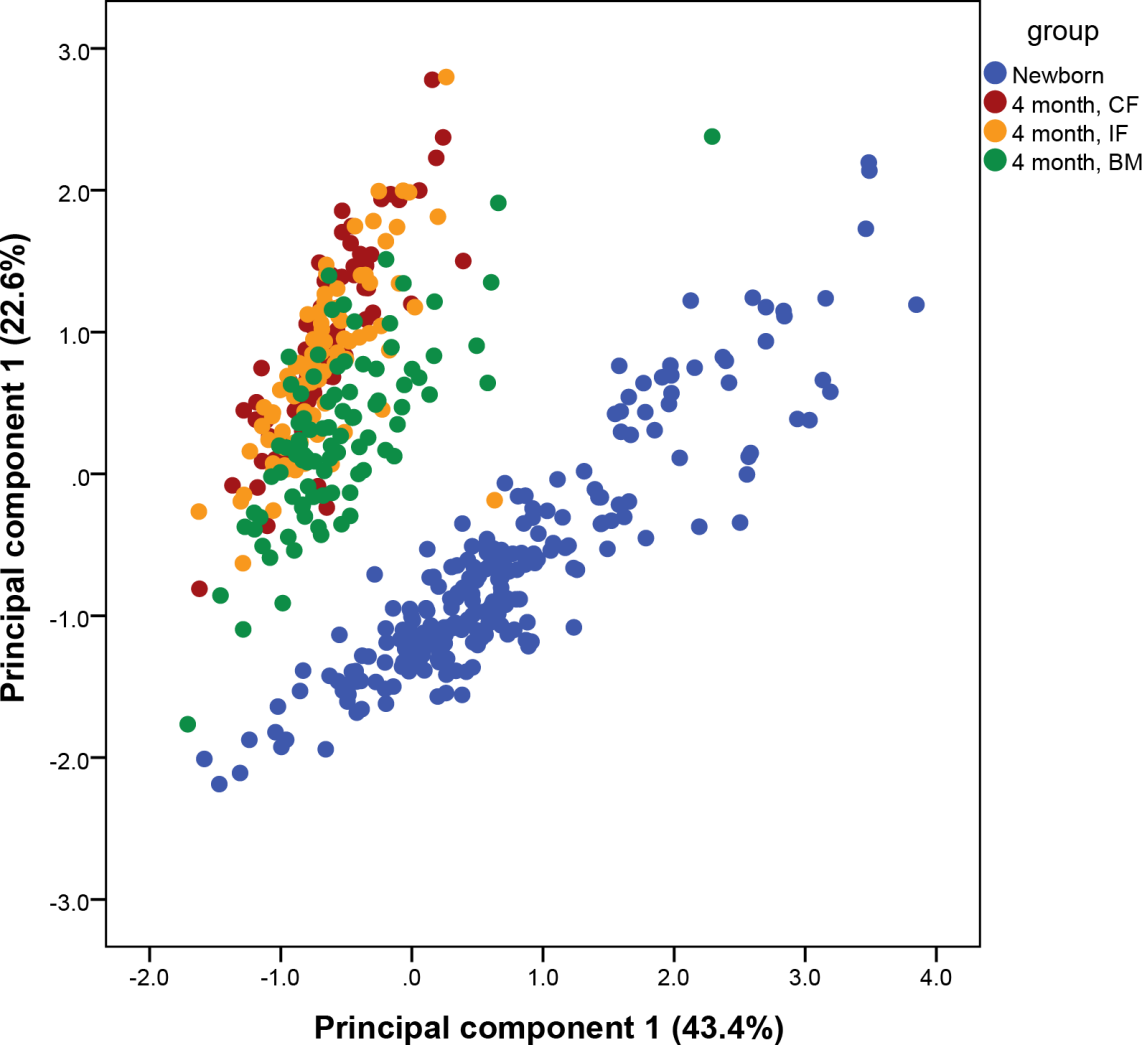
Principal component analyses of the phospholipid profile of newborn (n = 231) and at 4 month of age fed an intervention formula (IF, n = 83), a control formula (CF, n = 79) or breast milk (BM, n = 91).

**Fig 2:**
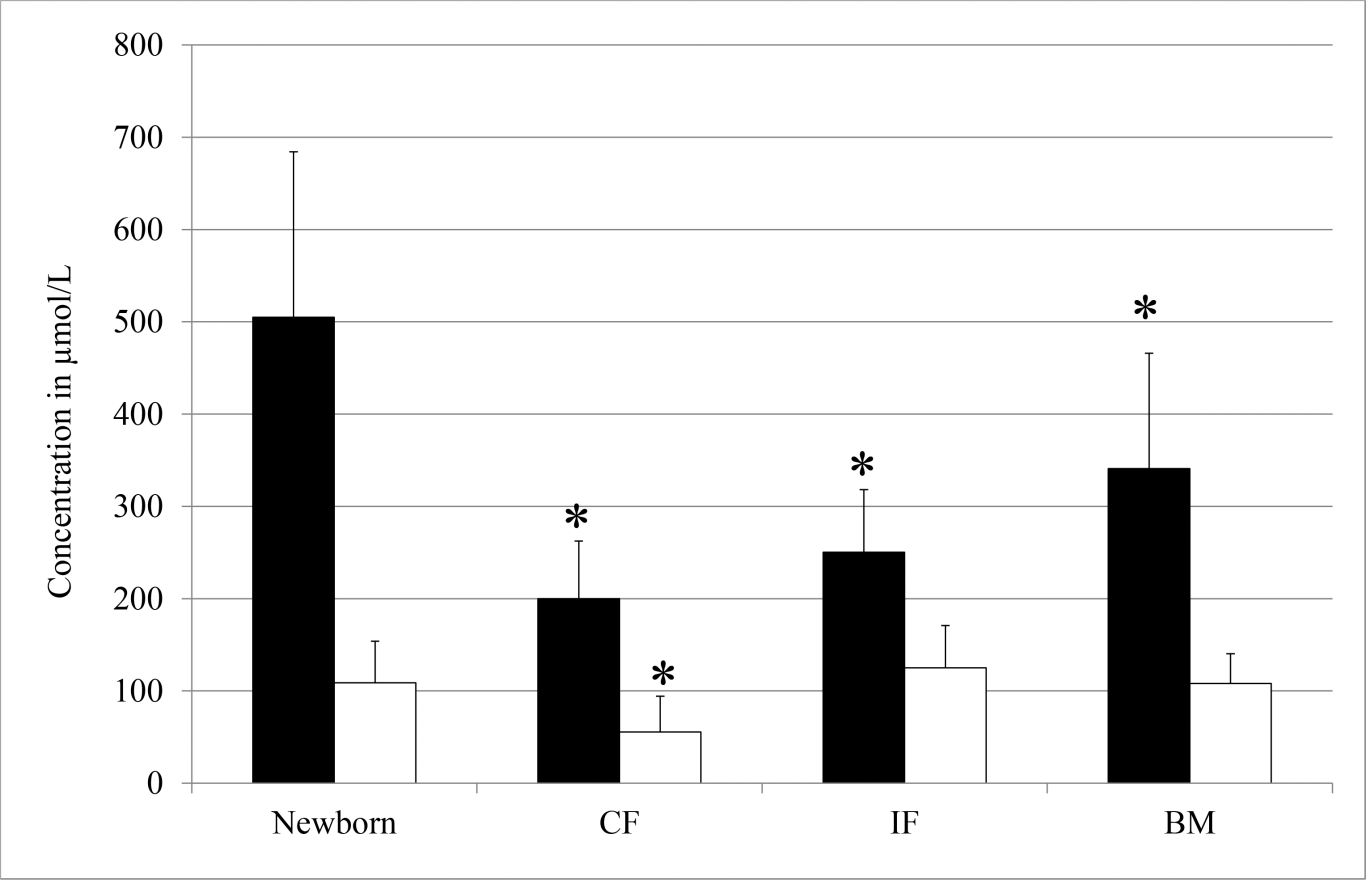
Sum of all species containing docosahexaenoic acid (black bar) and arachidonic acid (white bars) in newborn (n = 231) and at 4 month feeding a control formula (CF, n = 79), intervention formula (IF, n = 83) or breast milk (BM, n = 91). Significant differences to newborn are marked with an asterisk.

The plasma profile of newborn was strictly different from the plasma profile of 4 month old infants, irrespective of study group as illustrated in the plot of principal component 1 and 2 of the PCA analyses (Fig 1). The separation was found approximately along the angle bisector and thus both principal components were responsible for the separation. Loadings of the principal components did not identify a specific class of phospholipids, responsible for the separation. Mann-Whitney-U test identified 75 metabolites with different concentrations in the newborn compared to BM infants at 4 month of age (Table 2). This corresponds to 82% of all detected species. Most relevant different species were LPC16:1, LPC20:4, PC32:1, PC34:1 and PC36:4 with higher concentrations and LPC18:0, LPC18:2, PC32:2, PC36:2 and several PCe with lower concentrations in the newborn. The sum of all PCe species was rising from newborn to 4 month old infants with median (IQR) values of 85 (32) μmol/L for newborn, 92 (22) μmol/L for CF, 109 (24) μmol/L for IF and 127 (35) μmol/L for BM. PC36:4 was the highest phospholipid species in newborn, while PC34:2 was highest at 4 month of age. Strong effects were seen in species with AA and DHA, especially to the group of CF, which did not receive any additional AA and DHA over the study period. Nearly all species with these two fatty acids were affected and thus the sum was calculated to assess the total effect (Fig 2). Highest values for AA were found at the time of birth and all groups showed significant lower level at 4 month. Lowest values for AA as well as for DHA species were detected in the CF group. The DHA content at 4 month of age was not different between the IF and the BM group compared to the newborn.

**Table 2:** Median (M) and interquartile ranges (IQR) of plasma lipids (in μmol/L) of newborn (NB, n = 231) and at 4 month of age fed an intervention formula (IF, n = 83), a control formula (CF, n = 79) or breast milk (BM, n = 91). Mann-Whitney-U tests with Bonferroni correction were performed to compare between the groups. Significant (Sig.) changes (p <0.05) are marked in gray.

Metabolite	NB		CF		IF		BM		NB vs BM	CF vs IF	IF vs BM
	M	IQR	M	IQR	M	IQR	M	IQR			
LPC14:0	0.87	0.36	0.56	0.35	0.90	0.41	1.12	0.46	1.10E-11	3.67E-10	1.68E-05
LPC16:0	89.24	26.88	69.18	10.62	72.01	13.96	70.11	13.23	2.71E-14	3.43E-02	5.83E-02
LPC16:1	3.32	1.26	1.23	0.29	1.26	0.45	1.58	0.54	7.67E-38	9.67E-01	2.68E-07
LPC18:0	21.52	6.47	23.78	5.04	26.51	6.18	28.82	7.31	6.25E-27	2.37E-05	5.15E-04
LPC18:1	22.84	8.09	20.26	4.66	19.65	4.38	18.79	6.35	5.15E-09	4.79E-02	6.59E-01
LPC18:2	21.32	10.93	41.57	8.54	44.14	16.21	44.31	12.85	3.33E-33	1.01E-01	6.33E-01
LPC20:3	1.95	0.97	1.32	0.51	1.22	0.44	1.67	0.74	6.51E-04	1.49E-01	8.14E-10
LPC20:4	14.93	4.29	3.80	1.65	5.03	2.00	6.82	2.85	5.44E-38	4.47E-07	3.90E-12
LPC22:6	2.96	1.13	0.95	0.66	2.22	1.02	2.00	0.65	1.88E-22	8.31E-20	3.09E-02
LPCe16:0	0.24	0.13	0.26	0.16	0.29	0.15	0.47	0.22	6.88E-31	1.34E-01	1.22E-11
LPCe18:0	0.49	0.20	0.51	0.20	0.54	0.28	1.01	0.49	5.31E-35	1.01E-01	4.08E-14
PC30:0	3.16	1.38	1.32	0.78	2.26	0.72	3.02	1.25	2.33E-01	1.13E-10	1.04E-09
PC32:0	28.19	10.91	13.17	3.82	14.58	3.25	15.87	4.97	1.46E-33	4.96E-03	5.92E-03
PC32:1	24.49	12.26	7.14	2.05	7.36	2.72	8.69	3.36	4.27E-42	4.07E-01	3.01E-04
PC32:2	0.79	0.64	1.33	0.58	1.79	0.75	1.69	0.97	8.42E-28	8.34E-08	7.01E-01
PC34:1	249.96	121.42	188.03	44.08	182.17	44.89	149.28	39.94	5.63E-34	2.50E-01	1.31E-07
PC34:2	246.66	128.86	440.37	86.72	468.43	115.00	413.68	113.86	1.19E-22	1.65E-01	1.18E-04
PC34:3	4.70	2.66	7.55	2.24	8.93	2.44	6.77	2.47	6.45E-14	9.36E-06	1.04E-10
PC34:4	0.46	0.21	0.41	0.24	0.61	0.27	0.71	0.31	5.86E-17	1.02E-08	3.35E-03
PC34:5	0.08	0.06	0.06	0.04	0.11	0.06	0.08	0.05	3.63E-01	8.19E-08	9.48E-05
PC36:0	1.90	1.01	1.24	0.86	2.59	0.80	2.32	0.72	3.61E-05	3.01E-19	3.58E-04
PC36:1	40.54	18.94	42.50	9.20	43.85	11.02	41.40	11.92	9.12E-01	4.81E-01	5.83E-02
PC36:2	129.68	69.80	267.34	50.27	284.88	72.88	289.96	88.42	1.76E-36	5.90E-02	5.60E-01
PC36:3	75.49	38.56	107.04	23.38	95.96	26.04	107.78	37.21	1.82E-13	1.17E-03	2.40E-03
PC36:4	284.46	108.48	103.66	31.36	130.38	34.52	162.10	55.63	8.04E-34	6.93E-08	2.24E-07
PC36:5	4.89	2.64	3.74	1.86	6.03	2.49	4.71	1.48	1.37E-01	8.36E-15	4.27E-08
PC36:6	0.20	0.11	0.17	0.12	0.38	0.18	0.31	0.11	1.42E-16	3.33E-16	4.31E-05
PC38:1	0.64	0.62	0.94	0.54	1.33	0.53	1.39	0.44	1.51E-23	5.43E-08	4.95E-01
PC38:3	20.81	10.54	22.64	6.36	22.46	7.22	26.03	10.32	1.85E-08	1.96E-01	1.79E-09
PC38:4	170.97	61.08	69.42	28.41	89.31	24.20	136.35	52.48	2.73E-13	7.78E-07	3.58E-15
PC38:5	33.74	13.94	28.93	8.75	30.59	8.54	35.08	12.30	3.20E-01	1.23E-01	1.79E-05
PC38:6	74.86	32.02	35.39	25.96	83.73	34.15	68.30	22.33	2.97E-04	2.47E-20	2.69E-06
PC40:4	2.89	1.35	2.51	0.72	2.30	0.65	3.20	1.22	1.42E-03	7.76E-03	4.90E-15
PC40:5	6.89	3.57	7.01	2.08	7.21	2.38	8.46	3.16	5.60E-07	9.75E-01	1.13E-06
PC40:6	22.92	10.53	12.51	9.33	28.19	11.06	25.11	8.05	1.62E-02	5.52E-19	6.15E-02
PC42:5	0.23	0.11	0.26	0.12	0.27	0.11	0.35	0.12	7.63E-19	3.22E-01	1.14E-08
PC44:12	0.73	0.33	0.94	0.26	1.14	0.33	1.04	0.29	1.11E-15	3.65E-08	2.55E-03
PCe32:0	3.34	1.30	3.07	0.78	2.97	0.72	3.11	0.76	1.92E-02	8.32E-01	2.33E-01
PCe32:1	3.15	1.19	2.07	0.50	2.39	0.65	2.40	0.70	5.08E-16	1.38E-05	6.73E-01
PCe32:2	0.50	0.20	0.25	0.12	0.30	0.12	0.30	0.16	7.48E-20	3.35E-04	1.04E-01
PCe34:0	1.03	0.42	0.63	0.31	0.67	0.42	1.25	0.60	1.15E-03	3.81E-01	5.39E-14
PCe34:1	5.43	2.49	7.06	1.83	6.87	1.79	6.86	1.72	2.03E-08	5.06E-01	6.52E-01
PCe34:2	3.89	1.52	7.08	1.78	6.90	1.80	6.32	1.77	4.00E-27	2.14E-01	3.69E-02
PCe34:3	2.34	1.32	4.53	1.07	5.08	1.52	4.17	1.38	5.34E-28	4.08E-02	2.11E-03
PCe34:4	0.08	0.05	0.09	0.04	0.13	0.06	0.12	0.05	6.39E-09	2.32E-07	9.13E-04
PCe36:0	0.71	0.30	0.53	0.20	0.60	0.16	0.65	0.22	2.21E-03	1.01E-03	1.46E-01
PCe36:1	1.64	0.73	1.76	0.59	1.93	0.71	2.87	1.16	9.72E-24	5.22E-02	5.03E-11
PCe36:2	2.86	1.37	6.26	1.57	6.56	1.66	8.90	4.09	3.90E-42	5.30E-02	5.35E-10
PCe36:3	1.88	0.78	4.64	1.22	4.40	1.24	4.51	1.29	1.48E-39	7.94E-02	6.61E-01
PCe36:4	12.50	4.97	10.09	2.72	11.72	3.01	13.59	4.41	1.74E-02	9.22E-06	2.03E-06
PCe36:5	10.01	4.39	6.61	2.26	8.99	2.57	11.24	3.14	2.93E-02	8.65E-11	2.90E-07
PCe36:6	0.08	0.13	0.11	0.16	0.19	0.14	0.19	0.13	1.40E-13	4.02E-06	9.12E-01
PCe38:0	0.73	0.37	0.52	0.35	1.07	0.44	1.06	0.44	1.78E-15	2.71E-19	6.90E-01
PCe38:4	9.43	3.38	7.42	2.23	8.75	2.45	13.60	6.24	7.46E-15	1.95E-05	1.69E-15
PCe38:5	9.82	4.04	13.63	3.98	16.25	3.43	20.14	6.28	5.68E-35	2.45E-07	6.93E-09
PCe38:6	4.52	1.94	3.71	1.98	6.40	1.36	7.09	1.56	1.82E-19	3.59E-17	4.51E-03
PCe40:0	2.93	1.29	2.39	1.31	4.10	1.27	3.88	1.18	1.00E-09	4.39E-16	3.06E-02
PCe40:1	0.90	0.33	0.67	0.38	1.22	0.40	1.14	0.39	4.12E-09	2.90E-16	1.02E-01
PCe40:4	1.39	0.57	1.22	0.37	1.33	0.40	1.84	0.74	1.82E-11	2.94E-03	3.03E-13
PCe40:5	1.70	0.63	2.17	0.77	2.57	0.83	3.53	1.23	1.50E-37	1.89E-05	4.83E-12
PCe40:6	1.95	0.73	2.19	1.03	3.58	0.75	4.15	1.32	1.94E-37	1.28E-15	4.53E-07
PCe42:2	0.18	0.11	0.21	0.10	0.25	0.09	0.26	0.08	7.90E-14	3.09E-05	7.69E-01
PCe42:5	0.68	0.31	0.87	0.35	1.09	0.29	1.17	0.41	2.56E-28	1.45E-07	3.67E-02
PCe42:6	0.70	0.29	0.73	0.32	0.98	0.27	1.10	0.38	7.20E-23	5.63E-10	1.25E-03
SM12:0	0.32	0.15	0.39	0.38	0.88	0.29	0.86	0.45	2.73E-37	2.13E-16	6.27E-01
SM14:0	6.87	2.48	15.82	4.80	15.23	3.08	14.86	4.36	1.03E-38	4.25E-01	6.81E-02
SM14:1	0.61	0.23	0.35	0.22	0.61	0.22	0.78	0.42	1.10E-11	5.36E-13	3.30E-07
SM15:0	3.83	1.60	5.85	1.56	5.68	1.26	5.95	1.44	3.96E-22	5.46E-02	1.75E-02
SM16:0	103.65	32.88	102.98	20.99	118.18	17.23	125.20	28.13	2.00E-08	1.04E-06	2.21E-02
SM16:1	17.21	5.52	11.47	2.59	12.70	2.23	14.61	4.04	6.21E-08	3.05E-05	7.94E-06
SM17:0	2.20	0.88	1.83	0.50	1.86	0.56	3.13	1.30	1.60E-13	8.78E-02	5.60E-16
SM18:0	33.43	11.19	21.16	5.56	24.28	5.03	27.88	8.52	1.02E-10	4.83E-04	1.10E-06
SM18:1	18.74	6.71	7.02	2.17	8.62	2.33	12.78	5.20	1.16E-25	7.92E-07	3.70E-13
SM18:2	0.55	0.26	0.48	0.18	0.60	0.23	1.07	0.56	7.32E-24	2.40E-05	1.85E-14
SM19:0	1.40	0.60	1.56	0.75	1.58	0.53	1.89	0.54	4.09E-13	2.70E-01	2.25E-04
SM20:0	22.49	9.77	33.96	10.91	34.92	9.33	30.67	9.99	1.98E-10	4.68E-01	1.14E-04
SM20:1	10.26	4.12	11.30	2.40	12.27	3.42	11.36	2.67	5.62E-02	9.00E-03	5.44E-03
SM21:0	1.75	0.75	5.52	2.01	4.95	1.36	4.27	1.58	1.86E-39	1.42E-02	6.24E-06
SM22:0	14.30	5.00	13.01	6.59	14.58	5.10	11.13	6.03	4.19E-11	1.38E-01	1.13E-06
SM22:1	17.68	7.51	25.20	6.47	28.00	6.51	26.01	6.62	3.65E-21	2.58E-02	1.81E-01
SM22:3	5.84	1.97	2.44	0.76	2.85	0.84	3.23	0.93	4.17E-33	1.23E-07	1.24E-02
SM22:4	0.43	0.18	0.24	0.10	0.36	0.15	0.36	0.12	1.45E-05	5.63E-10	4.41E-01
SM23:0	3.57	1.76	6.91	1.88	6.93	1.53	6.48	1.84	3.46E-30	4.40E-01	2.16E-02
SM24:0	14.78	5.14	10.65	2.55	12.22	2.29	12.00	3.59	1.18E-10	2.88E-06	4.97E-01
SM24:1	35.04	12.74	29.50	8.33	37.82	7.29	35.11	8.96	8.47E-01	1.48E-11	1.86E-01
SM24:2	22.31	8.13	14.63	4.33	17.10	3.85	21.45	7.84	3.49E-02	6.67E-08	3.60E-08
SM24:3	11.96	4.13	5.10	1.95	6.17	1.99	9.71	4.55	2.44E-11	5.92E-05	1.82E-15
SM24:5	3.95	1.55	1.82	1.41	4.06	1.48	3.38	1.16	1.16E-06	3.27E-20	6.93E-05
SM25:1	0.87	0.43	0.54	0.24	0.70	0.26	1.15	0.60	8.53E-08	3.03E-07	7.86E-13
SM13:0	0.13	0.06	0.13	0.08	0.16	0.09	0.23	0.13	7.62E-24	1.86E-05	7.81E-07
SM17:1	0.31	0.15	0.20	0.10	0.23	0.10	0.46	0.22	3.27E-12	1.62E-02	2.17E-16

### Group comparison

To find out what effect supplemented AA and DHA on different plasma phospholipid species might have at 4 month of age, a Mann-Whitney-U test between the two formula groups was performed. A total of 51 species turned out to be significant different, which corresponds to 56% of detected metabolites (Table 2). Phospholipid species containing 6 double bonds presumably represent the presence of DHA, which are in detail LPC22:6, PC36:6, PC38:6, PC40:6, PCe36:6, PCe38:6, PCe40:6, and PCe42:6. The concentrations of all 8 species were significant lower in CF. In IF fed infants, all di-acylated PC species were between 2.25 and 2.37 fold higher. PCe species were enriched between 1.34 and 1.75 fold. Phospholipids containing AA were presumably LPC20:4, PC34:4, PC36:4, PC38:4, PC40:4, PCe34:4, PCe36:4, PCe38:4, and PCe40:4. All but PC40:4 and PCe40:4 were higher in the IF group.

To evaluate compositional shifts, the relative distribution of all DHA and AA containing species was calculated, separately. In the CF, a higher compositional content of DHA was found for the species PCe38:6, PCe40:6 and PCe42:6 and a lower content was found for PC38:6. The composition of AA species was affected with higher values of PC34:4 and lower values of PC40:4.

## Discussion

The phospholipid profile of infants a few days after birth was quite different to the phospholipid profiles at 4 months of age, irrespective of the study group. We found remarkable high concentrations of LPC(20:4) and PC(36:4) in newborn. PC(36:4) is composed to the main extend of PC(16:0/20:4) [14], which represents the phospholipid species with the highest content of AA. AA is known to be preferably transported through the placenta, but the process of transport is not jet completely understood [15]. In a previous study we found a high correlation of cord blood AA to placental PC(16:0/20:4) content and we speculated that placental PC(16:0/20:4) might be the source of AA for the fetal development and that this molecule plays a major role in the transport mechanism [16]. In the current study we could now specify the AA content in the newborn metabolism to 9 different AA containing species with a high proportion in the molecular species of PC(16:0/20:4). Furthermore, the high concentration of PC(36:4) in newborn might fit to the theory that fatty acids are released as free fatty acids from the placenta to the fetal circulation and then are further metabolised to phospholipids to be protect from the placental reabsorption [17]. Our finding of high LPC(20:4) concentration are not easy understandable, since lyso-phospholipids are thought to be produced from phospholipases A1 or A2. In phospholipids the AA is located at the sn-2 position and thus a phospholipase A1 would be necessary to produce LPC(20:4), albeit the phospholipases A1 is rarely distributed. Thus, it seems to be unrealistic that a phospholipase is responsible for the LPC(20:4). A further possible candidate might be the lecithin-acyl-transferase (LCAT), which transfers a fatty acid from phospholipids to cholesterol and releases a lyso-phospholipid. The sn-1 activity of LCAT was found to be dependent on the fatty acid chain length of the fatty acid on position sn-1 with 50% sn-1 activity for PC(16:0/20:4) [18]. Thus, this might be a good candidate for LPC(20:4) source. However, a new developed transporter for lyso-phospholipids was discussed to be responsible for the transport of LPC(22:6) through the placenta [19]. One could speculate about this process being also responsible for the transport of AA to the fetal metabolism.

In case of no further nutritional supply of LC-PUFA, their concentration decrease after birth and remain constant at lower level after a period of less than 3 months as shown in a study on the ^13^C-content of plasma fatty acids over time [20]. At the age of 4 months, the lower concentrations of phospholipid species with AA or DHA represents the endogenous synthesis of AA and DHA from linoleic acid (18:2n-6) and alpha-linolenic acid (18:3n-3) of CF feeding without additional AA and DHA supply, respectively [21]. The comparison between CF and newborn allowed assessing roughly the contribution of placental transfer to AA and DHA content in comparison to endogenous production. Fig 2 shows the level of all AA and DHA species in newborn and CF. In CF, level were significant lower with values of 40% AA and 51% DHA at 4 month of age compared to newborn. Thus, we conclude that the contribution of placental transfer during pregnancy of AA and DHA must be more than 50%. During pregnancy, the placental contribution is presumably even higher, since the endogenous production is not fully developed.

Breast milk is very complex and can show very high variation in the content. Thus, effects can always be multifactorial and causal changes might be difficult. In vivo studies of AA and DHA can be affected by their precursors such as dihomo-γ-linolenic acid and eicosapentaenoic acid. In this study, the two formula groups have the same fatty acid composition up to additional AA and DHA in the intervention group and effects can be limited to these fatty acids. The comparison between the IF and CF group allowed to assess the effect of dietary LC-PUFA to the endogenously produced LC-PUFA in very similar feeding groups. In IF group, all AA and DHA containing species but PC40:4 were enriched with smaller differences in species containing AA, but higher increases in DHA containing species. The composition of DHA containing species was slightly different between the CF and the IF with accumulation of DHA in PCe in the CF. Thus, either endogenously produced DHA is preferably incorporated into PCe or PCe are preferred when DHA is low abundant to prevent from oxidation. Tracer-studies might be able to answer this question in future studies.

The phospholipid profile in newborn showed very low level (~50%) of LPC(18:2), PC(32:2), PC(34:2), PC(36:2) and SM(18:2) in comparison to 4 month old infants. All species presumably contain linoleic acid (18:2) which is the precursor of endogenously synthesised AA. AA and DHA can be converted from linoleic acid and alpha-linolenic acid (18:3), respectively by elongation and desaturation of the carbon chain [22]. Phospholipid species containing alpha-linolenic acid, which are PC(34:3), PC(36:3) and PC(38:3), were also lower (~70%) in newborn than in infants at 4 month of age, although with smaller deviation. The low abundance of phospholipid species containing 18:2 and 18:3 in newborn indicates a low placental supply, while AA and DHA were much higher abundant. Since the conversion rate of precursors (18:2 and 18:3) to AA and DHA is low in adults, the rate might be even lower in foetuses and infants [23]. High demands during late pregnancy for cognitive and visual development is ensured by the direct placental supply of high level of AA and DHA.

The total amount of PCe was increasing from newborn to 4 month old infants, regardless of the study group. Regarding to literature, PCe might be important for the healthy neuronal development and could serve as reservoir for LC-PUFA [24]. Our results showed a dietary effect on the PCe content and we speculate about positive long-term effects for cognitive functions. PCe contain high amounts of LC-PUFA and thus the quantity of PCe might be explained by the consumption of LC-PUFA. The CF did not obtain any additional AA or DHA, while the IF was supplemented with AA and DHA and breast milk presumably contain highest values of different LC-PUFA [25]. A suggested preferred incorporation into PCe was not detected. As shown in Table 3, IF fed infants showed lower percentages of DHA within PCe species than infants fed CF. Moreover, PCe species without AA or DHA, such as PCe38:0, PCe40:1 or PCe40:0 were also higher in the group of IF, in comparison to the CF group. Thus, the synthesis of all PCe was promoted by the consumption of the IF. Nevertheless, infants of the BM group showed the highest values of all PCe species, which emphasized the outstanding role of breast milk for infants in the context of the suggested healthy properties of PCe.

**Table 3:** Median values and interquartile ranges (IQR) in % of total arachidonic acid and docosahexaenoic acid species in infant plasma at 4 month of age. Significant changes between groups are calculated by Mann-Whitney-U tests after Bonferroni correction and marked in gray.

	Control formula (n = 79)		Intervention formula (n = 83)		p-value
	Median	IQR	Median	IQR	
Arachidonic acid containing species					
LPC20:4	1.90%	0.49%	1.99%	0.43%	6.96E-02
PC34:4	0.21%	0.07%	0.24%	0.09%	9.95E-05
PC36:4	51.41%	2.11%	51.55%	2.64%	2.26E-01
PC38:4	35.75%	2.47%	36.20%	2.58%	4.05E-01
PC40:4	1.30%	0.23%	0.94%	0.24%	9.21E-19
PCe34:4	0.05%	0.02%	0.05%	0.02%	2.74E-02
PCe36:4	4.99%	0.87%	4.69%	0.72%	2.13E-03
PCe38:4	3.64%	0.69%	3.53%	0.69%	2.55E-02
PCe40:4	0.61%	0.15%	0.53%	0.13%	7.63E-04
Docosahexaenoic acid containing species					
LPC22:6	1.62%	0.49%	1.70%	0.57%	2.49E-02
PC36:6	0.30%	0.16%	0.30%	0.12%	6.47E-01
PC38:6	63.65%	2.57%	66.08%	3.15%	5.88E-14
PC40:6	22.09%	1.72%	22.29%	1.93%	8.92E-01
PCe36:6	0.16%	0.23%	0.15%	0.11%	3.47E-01
PCe38:6	6.70%	1.27%	5.04%	1.22%	9.29E-15
PCe40:6	3.84%	1.10%	2.81%	0.85%	5.33E-16
PCe42:6	1.21%	0.41%	0.81%	0.33%	1.43E-17

For an optimal development of infants, breast feeding is assumed to be the best choice of nutrition during the first 6 months of life. To optimise infant formula fatty acid content, the analyses of molecular phospholipid species may help to investigate metabolic relations between nutrition and physiological outcomes. The plasma phospholipid profile of infants fed formula was separated from the BM infants by PCA. Thus, different diets are represented by the plasma phospholipid profile. The IF infants showed a phospholipid profile between BM and CF. This result was confirmed by the group comparison which showed that less species were different between the IF and BM infants, than between CF and BM. Our results showed that dietary availability of LC-PUFA has a huge impact on the plasma phospholipids pattern of infants. Several studies have shown that supplementation of infant formula did improve the LC-PUFA status in different compartments. Regarding literature of mature breast milk fatty acid composition of triacylglycerols, the concentration of AA (0.41 +/- 0.05%) is at least twice as high as DHA (0.18 +/- 0.02%) [25]. In our study, the IF was added by equal amounts (72 mg/ 100 mL) of AA and DHA. Plasma concentrations of phospholipids containing DHA were within the same region for infants receiving the IF than BM and thus the IF provided appropriate DHA to match breast milk (Fig 2). However, a concentration of 72 mg/100 mL AA seems not to be enough, compared to BM infants (Fig 2). To realize a fatty acid composition of infant formulas most closely to the breastmilk, a higher concentration of AA than 72 mg/ 100 ml should be considered.

A limitation of the study might be the missing of further interesting lipid fractions, such as phosphatidylethanolamines, cholesterol ester or triacylglycerols. Since the used method is a screening method for a serious of phospholipid species, there are limitations in quantification and identification. Identification occurred based on mass spectrometric detection and in some cases there might be more than one species behind the mass transition. Identification occurred to our best knowledge for the highest concentrated species. A further limitation might be the correction of PCe and SM by internal standard of PC(28:0), which might not compensate for all class specific effects during sample preparation and ionisation. However, we belief that PC(28:0) might be suitable for compensating the overwhelming class of effects because of very similar chemical constitution of PC, PCe and SM. Quantification was applied including isotopomer correction, which can result in higher variation of low concentrated species. Isotopomer correction was made until M+4 isotopomers to ensure that no false positive results were obtained.

## Conclusion

In conclusion, we have shown that infant plasma phospholipid profile changes from newborn to 4 month old infants and is dependent on the dietary fatty acid composition. Infants of CF group, which did not obtain additional LC-PUFA showed values of 40% (AA) and 51% (DHA) in comparison to newborn. The supplementation of an infant formula with AA and DHA resulted in markedly increased related phospholipid species. DHA was enriched in PCe of CF fed infants, probably to save residual DHA from oxidation, while PCe synthesis may be enhanced by PUFA supply in breast fed and IF infants.

## Supporting Information

S1 File Method description. (PDF)

## Abbreviations

PCe: ether-linked phosphatidylcholines
AA: arachidonic acid
BM: breastmilk
CF: control formula
PC: diacyl-phosphatidylcholines
DHA: docosahexaenoic acid
IQR: interquartile ranges
IF: intervention formula
LC-PUFA: long-chain polyunsaturated fatty acids
LPC: lyso-phosphatidylcholines
PCA: principal component analyses
SM: sphingomyelines

## References

[1] Koletzko B, Baker S, Cleghorn G, Neto UF, Gopalan S, Hernell O, et al Global standard for the composition of infant formula: recommendations of an ESPGHAN coordinated international expert group. Journal of pediatric gastroenterology and nutrition. 2005;41:584–99. 16254515 10.1097/01.mpg.0000187817.38836.42

[2] The Commission of the European Communities. Commission Directive 2006/141/EC of 22 December 2006 on infant formulae and amending Directive 1999/21/EC. Official Journal of the European Union. 2006;L401:1–33.

[3] Makrides M, Uauy R. LCPUFAs as conditionally essential nutrients for very low birth weight and low birth weight infants: metabolic, functional, and clinical outcomes-how much is enough? Clinics in perinatology. 2014;41:451–61. 10.1016/j.clp.2014.02.012 24873843

[4] Fekete K, Marosvolgyi T, Jakobik V, Decsi T. Methods of assessment of n-3 long-chain polyunsaturated fatty acid status in humans: a systematic review. The American journal of clinical nutrition. 2009;89:2070S–84S. 10.3945/ajcn.2009.27230I 19420097

[5] Kuratko CN, Salem N Jr. Biomarkers of DHA status. Prostaglandins, leukotrienes, and essential fatty acids. 2009;81:111–8. 10.1016/j.plefa.2009.05.007 19545987

[6] Dowhan W. Molecular basis for membrane phospholipid diversity: why are there so many lipids? Annual review of biochemistry. 1997;66:199–232. 9242906 10.1146/annurev.biochem.66.1.199

[7] Fleddermann M, Demmelmair H, Grote V, Nikolic T, Trisic B, Koletzko B. Infant formula composition affects energetic efficiency for growth: the BeMIM study, a randomized controlled trial. Clinical nutrition. 2014;33:588–95. 10.1016/j.clnu.2013.12.007 24411489

[8] Hodson L, Skeaff CM, Fielding BA. Fatty acid composition of adipose tissue and blood in humans and its use as a biomarker of dietary intake. Progress in lipid research. 2008;47:348–80. 10.1016/j.plipres.2008.03.003 18435934

[9] Rauschert S, Uhl O, Koletzko B, Kirchberg F, Mori TA, Huang RC, et al Lipidomics reveals associations of phospholipids with obesity and insulin resistance in young adults. J Clin Endocrinol Metab. 2015:jc20153525. 10.1210/jc.2015-3525 26709969

[10] Lindsay KL, Hellmuth C, Uhl O, Buss C, Wadhwa PD, Koletzko B, et al Longitudinal Metabolomic Profiling of Amino Acids and Lipids across Healthy Pregnancy. PLoS One. 2015;10:e0145794 10.1371/journal.pone.0145794 26716698 PMC4699222

[11] Larque E, Demmelmair H, Gil-Sanchez A, Prieto-Sanchez MT, Blanco JE, Pagan A, et al Placental transfer of fatty acids and fetal implications. The American journal of clinical nutrition. 2011;94:1908S–13S. 10.3945/ajcn.110.001230 21562082

[12] Fahy E, Subramaniam S, Murphy RC, Nishijima M, Raetz CR, Shimizu T, et al Update of the LIPID MAPS comprehensive classification system for lipids. Journal of lipid research. 2009;50 Suppl:S9–14. 10.1194/jlr.R800095-JLR200 19098281 PMC2674711

[13] Wishart DS, Jewison T, Guo AC, Wilson M, Knox C, Liu Y, et al HMDB 3.0—The Human Metabolome Database in 2013. Nucleic acids research. 2013;41:D801–7. 10.1093/nar/gks1065 23161693 PMC3531200

[14] Uhl O, Glaser C, Demmelmair H, Koletzko B. Reversed phase LC/MS/MS method for targeted quantification of glycerophospholipid molecular species in plasma. Journal of chromatography B, Analytical technologies in the biomedical and life sciences. 2011;879:3556–64. 10.1016/j.jchromb.2011.09.043 22014895

[15] Koletzko B, Larque E, Demmelmair H. Placental transfer of long-chain polyunsaturated fatty acids (LC-PUFA). Journal of perinatal medicine. 2007;35 Suppl 1:S5–11. 17302540 10.1515/JPM.2007.030

[16] Uhl O, Demmelmair H, Segura MT, Florido J, Rueda R, Campoy C, et al Effects of obesity and gestational diabetes mellitus on placental phospholipids. Diabetes research and clinical practice. 2015;109:364–71. 10.1016/j.diabres.2015.05.032 26021978

[17] Haggarty P. Fatty acid supply to the human fetus. Annual review of nutrition. 2010;30:237–55. 10.1146/annurev.nutr.012809.104742 20438366

[18] Subbaiah PV, Liu M, Paltauf F. Role of sn-2 acyl group of phosphatidylcholine in determining the positional specificity of lecithin-cholesterol acyltransferase. Biochemistry. 1994;33:13259–66. 7947733 10.1021/bi00249a012

[19] Prieto-Sanchez MT, Ruiz-Palacios M, Blanco-Carnero JE, Pagan A, Hellmuth C, Uhl O, et al Placental MFSD2a transporter is related to decreased DHA in cord blood of women with treated gestational diabetes. Clinical nutrition. 2016. 10.1016/j.clnu.2016.01.014 26869380

[20] Carnielli VP, Simonato M, Verlato G, Luijendijk I, De Curtis M, Sauer PJ, et al Synthesis of long-chain polyunsaturated fatty acids in preterm newborns fed formula with long-chain polyunsaturated fatty acids. The American journal of clinical nutrition. 2007;86:1323–30. 17991642 10.1093/ajcn/86.5.1323

[21] Glaser C, Heinrich J, Koletzko B. Role of FADS1 and FADS2 polymorphisms in polyunsaturated fatty acid metabolism. Metabolism: clinical and experimental. 2010;59:993–9. 20045144 10.1016/j.metabol.2009.10.022

[22] Gibson RA, Muhlhausler B, Makrides M. Conversion of linoleic acid and alpha-linolenic acid to long-chain polyunsaturated fatty acids (LCPUFAs), with a focus on pregnancy, lactation and the first 2 years of life. Maternal & child nutrition. 2011;7 Suppl 2:17–26. 21366864 10.1111/j.1740-8709.2011.00299.x PMC6860743

[23] Koletzko B, Lien E, Agostoni C, Bohles H, Campoy C, Cetin I, et al The roles of long-chain polyunsaturated fatty acids in pregnancy, lactation and infancy: review of current knowledge and consensus recommendations. Journal of perinatal medicine. 2008;36:5–14. 10.1515/JPM.2008.001 18184094

[24] Braverman NE, Moser AB. Functions of plasmalogen lipids in health and disease. Biochimica et biophysica acta. 2012;1822:1442–52. 10.1016/j.bbadis.2012.05.008 22627108

[25] Sala-Vila A, Castellote AI, Rodriguez-Palmero M, Campoy C, Lopez-Sabater MC. Lipid composition in human breast milk from Granada (Spain): changes during lactation. Nutrition. 2005;21:467–73. 15811767 10.1016/j.nut.2004.08.020

