# Supplementary material for: Phospholipid Species in Newborn and 4 Month Old Infants after Consumption of Different Formulas or Breast Milk
Source: PLoS One. 2016 Aug 29;11(8):e0162040. doi: 10.1371/journal.pone.0162040 (PMC5003354; doi:10.1371/journal.pone.0162040)
Supplement: S1 File — (PDF) [file pone.0162040.s001.pdf]

Method description

Chemicals requirements

- Control Plasma (CP), Level 1 & Level 2, Recipe
- 1-tridecanoyl-2-hydroxy-sn-glycero-3-phosphocholine, LPC(13:0), Avanti Polar Lipids (855476P)
- 1,2-dimyristoyl-sn-glycero-3-phosphocholine, PC(28:0), Avanti Polar Lipids (850345P)
- D3-Carnitine C2, Cambridge Isotope Laboratories (DLM-754-PK)
- D3-Carnitine C8, Cambridge Isotope Laboratories (DLM-755-0.01)
- D3-Carnitine C16, Cambridge Isotope Laboratories (DLM-1263-0.01)
- 13C6-D-Glucose, Cambridge Isotope Laboratories (CLM-1396)
- Riplate, 1.2 mL 96-deepwell, Carl Roth GmbH + Co. KG (EN06.1)
- 96 deepwell-plate, 1.0 mL, polypropylene, Agilent (5042-6454)
- Ammonium acetate, Fluka (73594-25G-F)
- Water, HPLC grade, J.T. Backer
- Methanol, HPLC grade, CLN GmbH (CLN-8402.2500)
- Isopropanol, HPLC grade, LGC Standards (SO-3043-B040)

Sample preparation

Proteins of 10 µL plasma were precipitated by adding 500 µL methanol, including 1-tridecanoyl-2-hydroxy-sn-glycero-3-phosphocholine and 1,2-dimyristoyl-sn-glycero-3-phosphocholine as internal standards with a concentration of approximately 2 µmol/L and ammonium acetate with a concentration of 0.4 g/l. Samples were prepared using a 1.2 mL 96-deepwell plate. The plate was shaked for 30 minutes with 800 rpm and afterwards cooled for 20 minutes at -20°C. After centrifugation at 4000 rpm for 10 minutes 100 µL of the supernatant were transferred in a 1.0 mL 96 deepwell-plate, prefilled with 350 µL methanol.

Mass spectrometric analyses

After centrifugation, supernatants were used for flow-injection mass spectrometry analysis. The liquid chromatographic system (Agilent, Waldbronn, Germany) was coupled to a triple quadrupole mass spectrometer (QTRAP4000, Sciex, Darmstadt, Germany) with an electrospray ionization source. Analyses were split in two periods per sample each with 30 µL injection volume to cover the full range of analytes. Samples were injected to 40 µL/min mobile phase with isocratic elution (76% isopropanol, 19% methanol and 5% water) which results in broader peak elution than normal flow-injection analyses to ensure enough data points for the mass spectrometry analyses. Low flow rates were found to reduce lifetime of ESI needles, which would affect reproducibility and thus the flow was diluted by adding a further 200 µL/min mobile phase "post-column" via T-piece adapter to stabilize the electro spray for ionization. Mass spectrometric analysis was run in Multiple Reaction Monitoring mode. Period one was run with positive and negative ionisation and period two was run with positive ionisation.

Quantification

Quantification of metabolites has been done by comparison of signal-to-internal standard-ratios between samples and commercial available lyophilized aliquots of control plasma (Recipe, Germany). The concentrations of the control plasma were determined by AbsoluteIDQ p150 Kit from Biocrates®, a previous published LC-MS/MS method (Uhl, JChormB, 2011) and by in-house quantification with various standards. The entire analytical process was post-processed by Analyst 1.5.1 and the isotopomer correction for up to M+4 was applied by R (programming language, version 3.0.1).

Periode 1 – positive

Q1	Q3	time	DP	EP	CE	CXP
162.1	85.1	20	61	10	27	15
204.1	85.1	20	41	10	27	15
206.1	85.1	20	41	10	27	15
207.1	85.1	50	41	10	27	15
216.1	85.1	20	49	10	27	15
218.1	85.1	20	46	10	29	15
230.1	85.1	20	52	10	29	15
232.1	85.1	20	46	10	29	15
234.1	85.1	20	53	10	30	15
244.1	85.1	20	55	10	31	15
246.1	85.1	20	46	10	29	15
248.1	85.1	20	55	10	32	15
258.1	85.1	20	57	10	33	15
260.1	85.1	20	56	10	27	15
262.1	85.1	20	58	10	33	15
274.1	85.1	20	60	10	35	15
276.2	85.1	20	61	10	35	15
286.2	85.1	20	63	10	37	15
288.2	85.1	20	66	10	33	15
290.2	85.1	20	63	10	37	15
291.2	85.1	50	66	10	33	15
302.2	85.1	20	66	10	39	15
304.2	85.1	20	66	10	39	15
312.2	85.1	20	67	10	40	15
314.2	85.1	20	68	10	40	15
316.2	85.1	20	56	10	37	15
342.3	85.1	20	73	10	44	15
344.3	85.1	20	73	10	44	15
368.3	85.1	20	78	10	47	15
370.3	85.1	20	78	10	47	15
372.3	85.1	20	86	10	45	15
374.3	85.1	20	86	10	45	15
384.3	85.1	20	81	10	49	15
386.3	85.1	20	81	10	50	15
396.3	85.1	20	83	10	51	15
398.3	85.1	20	84	10	51	15
400.3	85.1	20	84	10	51	15
402.3	85.1	20	84	10	51	15
403.3	85.1	50	84	10	51	15
412.3	85.1	20	86	10	53	15

414.3	85.1	20	87	10	53	15
416.3	85.1	20	87	10	53	15
420.3	85.1	20	89	10	54	15
422.3	85.1	20	89	10	54	15
424.3	85.1	20	89	10	54	15
426.3	85.1	20	89	10	55	15
428.3	85.1	20	94	10	63	15
440.3	85.1	20	94	10	65	15
442.3	85.1	20	94	10	65	15
446.3	85.1	20	96	10	65	15
448.3	85.1	20	96	10	65	15
450.3	85.1	20	96	10	65	15
452.3	85.1	20	96	10	65	15
454.3	85.1	20	96	10	65	15
456.3	85.1	20	96	10	65	15
472.3	85.1	20	96	10	65	15
474.3	85.1	20	96	10	65	15
476.3	85.1	20	96	10	65	15
478.3	85.1	20	96	10	65	15
480.3	85.1	20	96	10	65	15
482.3	85.1	20	96	10	65	15
484.3	85.1	20	96	10	65	15
449.3	184	20	79	10	30	10
450.3	184	20	79	10	30	10
451.3	184	20	79	10	30	10
452.3	184	20	79	10	30	10
453.3	184	20	79	10	30	10
454.3	184	50	79	10	30	10
455.3	184	20	79	10	30	10
456.3	184	20	79	10	30	10
457.3	184	20	79	10	30	10
458.3	184	20	79	10	30	10
459.3	184	20	79	10	30	10
460.3	184	20	79	10	30	10
461.3	184	20	79	10	30	10
462.3	184	20	79	10	30	10
463.3	184	20	79	10	30	10
464.3	184	20	79	10	30	10
465.3	184	20	79	10	30	10
466.3	184	20	79	10	30	10
467.3	184	20	79	10	30	10
468.3	184	20	79	10	30	10
469.3	184	20	79	10	30	10
470.3	184	20	79	10	30	10

471.3	184	20	79	10	30	10
472.3	184	20	79	10	30	10
473.3	184	20	79	10	30	10
474.3	184	20	79	10	30	10
475.3	184	20	79	10	30	10
476.3	184	20	79	10	30	10
477.3	184	20	79	10	30	10
478.3	184	20	79	10	30	10
479.3	184	20	79	10	30	10
480.3	184	20	79	10	30	10
481.3	184	20	79	10	30	10
482.3	184	20	79	10	30	10
483.3	184	20	79	10	30	10
484.3	184	20	79	10	30	10
485.3	184	20	79	10	30	10
486.3	184	20	79	10	30	10
487.3	184	20	79	10	30	10
488.3	184	20	79	10	30	10
489.3	184	20	79	10	30	10
490.3	184	20	79	10	30	10
491.3	184	20	79	10	30	10
492.3	184	20	79	10	30	10
493.3	184	20	79	10	30	10
494.3	184	20	79	10	30	10
495.3	184	20	79	10	30	10
496.3	184	20	81	10	31	10
497.3	184	20	81	10	31	10
498.3	184	20	81	10	31	10
499.3	184	20	81	10	31	10
500.3	184	20	81	10	31	10
501.3	184	20	81	10	31	10
502.3	184	20	81	10	31	10
503.3	184	20	81	10	31	10
504.3	184	20	81	10	31	10
505.3	184	20	81	10	31	10
506.3	184	20	81	10	31	10
507.3	184	20	81	10	31	10
508.3	184	20	81	10	31	10
509.3	184	20	81	10	31	10
510.3	184	20	82	10	31	10
511.3	184	20	82	10	31	10
512.3	184	20	82	10	31	10
513.3	184	20	82	10	31	10
514.3	184	20	82	10	31	10

515.3	184	20	82	10	31	10
516.3	184	20	82	10	31	10
517.3	184	20	82	10	31	10
518.3	184	20	82	10	31	10
519.3	184	20	82	10	31	10
520.3	184	20	82	10	31	10
521.3	184	20	82	10	31	10
522.3	184	20	82	10	31	10
523.3	184	20	82	10	31	10
524.3	184	20	85	10	31	10
525.3	184	20	85	10	31	10
526.3	184	20	85	10	31	10
527.3	184	20	85	10	31	10
528.3	184	20	85	10	31	10
529.3	184	20	85	10	31	10
530.3	184	20	85	10	31	10
531.3	184	20	85	10	31	10
532.3	184	20	85	10	31	10
533.3	184	20	85	10	31	10
534.3	184	20	85	10	31	10
535.3	184	20	85	10	31	10
536.3	184	20	85	10	31	10
537.3	184	20	85	10	31	10
538.3	184	20	85	10	31	10
539.3	184	20	85	10	31	10
540.3	184	20	85	10	31	10
541.3	184	20	85	10	31	10
542.3	184	20	85	10	31	10
543.3	184	20	85	10	31	10
544.3	184	20	85	10	31	10
545.3	184	20	85	10	31	10
546.3	184	20	87	10	31	10
547.3	184	20	87	10	31	10
548.3	184	20	87	10	31	10
549.3	184	20	87	10	31	10
550.3	184	20	87	10	31	10
551.3	184	20	87	10	31	10
552.3	184	20	87	10	31	10
553.3	184	20	87	10	31	10
554.3	184	20	87	10	31	10
555.3	184	20	87	10	31	10
556.3	184	20	87	10	31	10
557.3	184	20	87	10	31	10
558.3	184	20	87	10	31	10

559.3	184	20	87	10	31	10
560.3	184	20	87	10	31	10
561.3	184	20	87	10	31	10
562.3	184	20	87	10	31	10
563.3	184	20	87	10	31	10
564.3	184	20	87	10	31	10
565.3	184	20	87	10	31	10
566.3	184	20	87	10	31	10
567.4	184	20	94	10	32	10
568.4	184	20	94	10	32	10
569.4	184	20	94	10	32	10
570.4	184	20	94	10	32	10
571.4	184	20	94	10	32	10
572.4	184	20	94	10	32	10
573.4	184	20	94	10	32	10
574.4	184	20	94	10	32	10
575.4	184	20	94	10	32	10
576.4	184	20	94	10	32	10
577.4	184	20	94	10	32	10
578.4	184	20	94	10	32	10
579.4	184	20	94	10	32	10
580.4	184	20	94	10	32	10

Period 1 – negative

Q1	Q3	time	DP	EP	CE	CXP
179.05	89	50	-55	-10	-12	-15
185.05	92	50	-55	-10	-12	-15

Period 2 – positive

Q1	Q3	time	DP	EP	CE	CXP
581.4	184	20	94	10	32	10
582.4	184	20	94	10	32	10
583.4	184	20	94	10	32	10
584.4	184	20	94	10	32	10
585.4	184	20	94	10	32	10
586.4	184	20	94	10	32	10
587.4	184	20	94	10	32	10
588.4	184	20	94	10	32	10
589.4	184	20	94	10	32	10

590.4	184	20	94	10	32	10
591.4	184	20	94	10	32	10
592.4	184	20	94	10	32	10
593.4	184	20	94	10	32	10
594.4	184	20	94	10	32	10
595.4	184	20	94	10	32	10
596.4	184	20	94	10	32	10
597.4	184	20	94	10	32	10
598.4	184	20	94	10	32	10
599.4	184	20	94	10	32	10
600.4	184	20	94	10	32	10
601.4	184	20	94	10	32	10
602.4	184	20	94	10	32	10
603.4	184	20	94	10	32	10
604.4	184	20	94	10	32	10
605.4	184	20	94	10	32	10
606.4	184	20	94	10	32	10
607.4	184	20	94	10	32	10
608.4	184	20	94	10	32	10
609.4	184	20	94	10	32	10
610.4	184	20	94	10	32	10
611.4	184	20	94	10	32	10
612.4	184	20	94	10	32	10
613.4	184	20	94	10	32	10
614.4	184	20	94	10	32	10
615.4	184	20	94	10	32	10
616.4	184	20	94	10	32	10
617.4	184	20	94	10	32	10
618.4	184	20	94	10	32	10
619.4	184	20	94	10	32	10
620.4	184	20	94	10	32	10
621.4	184	20	96	10	33	10
622.4	184	20	98	10	33	10
623.4	184	20	98	10	33	10
624.4	184	20	98	10	33	10
625.4	184	20	98	10	33	10
626.4	184	20	98	10	33	10
627.4	184	20	98	10	33	10
628.4	184	20	98	10	33	10
629.4	184	20	98	10	33	10
630.4	184	20	98	10	33	10
631.4	184	20	98	10	33	10
632.4	184	20	98	10	33	10
633.4	184	20	98	10	33	10

634.4	184	20	98	10	33	10
635.4	184	20	98	10	33	10
636.5	184	20	101	10	34	10
637.5	184	20	101	10	34	10
638.5	184	20	101	10	34	10
639.5	184	20	101	10	34	10
640.5	184	20	101	10	34	10
641.5	184	20	101	10	34	10
642.5	184	20	101	10	34	10
643.5	184	20	101	10	34	10
644.5	184	20	101	10	34	10
645.5	184	20	101	10	34	10
646.5	184	20	101	10	34	10
647.5	184	20	101	10	34	10
648.5	184	20	101	10	34	10
649.5	184	20	101	10	34	10
650.5	184	20	103	10	35	10
651.5	184	20	103	10	35	10
652.5	184	20	103	10	35	10
653.5	184	20	103	10	35	10
654.5	184	20	103	10	35	10
655.5	184	20	103	10	35	10
656.5	184	20	103	10	35	10
657.5	184	20	103	10	35	10
658.5	184	20	103	10	35	10
659.5	184	20	103	10	35	10
660.5	184	20	103	10	35	10
661.5	184	20	103	10	35	10
662.5	184	20	103	10	35	10
663.5	184	20	103	10	35	10
664.5	184	20	105	10	35	10
665.5	184	20	105	10	35	10
666.5	184	20	105	10	35	10
667.5	184	20	105	10	35	10
668.5	184	20	105	10	35	10
669.5	184	20	105	10	35	10
670.5	184	20	105	10	35	10
671.5	184	20	105	10	35	10
672.5	184	20	105	10	35	10
673.5	184	20	105	10	35	10
674.5	184	20	105	10	35	10
675.5	184	20	105	10	35	10
676.5	184	20	106	10	35	10
677.5	184	20	106	10	35	10

678.5	184	50	108	10	36	10
679.5	184	20	108	10	36	10
680.5	184	20	108	10	36	10
681.5	184	20	108	10	36	10
682.5	184	20	108	10	36	10
683.5	184	20	108	10	36	10
684.5	184	20	108	10	36	10
685.5	184	20	108	10	36	10
686.5	184	20	108	10	36	10
687.5	184	20	108	10	36	10
688.5	184	20	108	10	36	10
689.5	184	20	108	10	36	10
690.5	184	20	109	10	36	10
691.5	184	20	109	10	36	10
692.5	184	20	109	10	36	10
693.5	184	20	109	10	36	10
694.5	184	20	109	10	36	10
695.5	184	20	109	10	36	10
696.5	184	20	109	10	36	10
697.5	184	20	109	10	36	10
698.5	184	20	109	10	36	10
699.5	184	20	109	10	36	10
700.5	184	20	109	10	36	10
701.5	184	20	111	10	37	10
702.5	184	20	111	10	37	10
703.5	184	20	112	10	37	10
704.5	184	20	112	10	37	10
705.5	184	20	112	10	37	10
706.5	184	20	114	10	37	10
707.5	184	20	114	10	37	10
708.5	184	20	114	10	37	10
709.5	184	20	114	10	37	10
710.5	184	20	114	10	37	10
711.5	184	20	114	10	37	10
712.5	184	20	114	10	37	10
713.5	184	20	114	10	37	10
714.5	184	20	114	10	37	10
715.5	184	20	114	10	37	10
716.5	184	20	114	10	37	10
717.5	184	20	114	10	38	10
718.5	184	20	115	10	38	10
719.5	184	20	115	10	38	10
720.5	184	20	115	10	38	10
721.5	184	20	115	10	38	10

722.5	184	20	115	10	38	10
723.5	184	20	115	10	38	10
724.5	184	20	115	10	38	10
725.6	184	20	115	10	38	10
726.6	184	20	115	10	38	10
727.6	184	20	115	10	38	10
728.6	184	20	117	10	38	10
729.6	184	20	117	10	38	10
730.6	184	20	117	10	38	10
731.6	184	20	117	10	38	10
732.6	184	20	118	10	39	10
733.6	184	20	118	10	39	10
734.6	184	20	119	10	39	10
735.6	184	20	119	10	39	10
736.6	184	20	119	10	39	10
737.6	184	20	119	10	39	10
738.6	184	20	119	10	39	10
739.6	184	20	119	10	39	10
740.6	184	20	119	10	39	10
741.6	184	20	119	10	39	10
742.6	184	20	120	10	39	10
743.6	184	20	120	10	39	10
744.6	184	20	120	10	39	10
745.6	184	20	120	10	39	10
746.6	184	20	121	10	39	10
747.6	184	20	121	10	39	10
748.6	184	20	122	10	40	10
749.6	184	20	122	10	40	10
750.6	184	20	122	10	40	10
751.6	184	20	122	10	40	10
752.6	184	20	122	10	40	10
753.6	184	20	122	10	40	10
754.6	184	20	122	10	40	10
755.6	184	20	123	10	40	10
756.6	184	20	123	10	40	10
757.6	184	20	123	10	40	10
758.6	184	20	123	10	40	10
759.6	184	20	123	10	40	10
760.6	184	20	125	10	41	10
761.6	184	20	125	10	41	10
762.6	184	20	125	10	41	10
763.6	184	20	125	10	41	10
764.6	184	20	125	10	41	10
765.6	184	20	125	10	41	10

766.6	184	20	125	10	41	10
767.6	184	20	125	10	41	10
768.6	184	20	126	10	41	10
769.6	184	20	126	10	41	10
770.6	184	20	126	10	41	10
771.6	184	20	126	10	41	10
772.6	184	20	127	10	41	10
773.6	184	20	127	10	41	10
774.6	184	20	127	10	41	10
775.6	184	20	127	10	41	10
776.6	184	20	128	10	42	10
777.6	184	20	128	10	42	10
778.6	184	20	128	10	42	10
779.6	184	20	128	10	42	10
780.6	184	20	128	10	42	10
781.6	184	20	129	10	42	10
782.6	184	20	129	10	42	10
783.6	184	20	129	10	42	10
784.6	184	20	130	10	42	10
785.6	184	20	130	10	42	10
786.6	184	20	130	10	42	10
787.6	184	20	130	10	42	10
788.6	184	20	131	10	43	10
789.6	184	20	131	10	43	10
790.6	184	20	131	10	43	10
791.6	184	20	131	10	43	10
792.6	184	20	132	10	43	10
793.6	184	20	132	10	43	10
794.6	184	20	132	10	43	10
795.6	184	20	132	10	43	10
796.6	184	20	133	10	43	10
797.6	184	20	133	10	43	10
798.6	184	20	133	10	43	10
799.6	184	20	133	10	43	10
800.6	184	20	133	10	43	10
801.6	184	20	134	10	43	10
802.6	184	20	134	10	44	10
803.6	184	20	134	10	44	10
804.6	184	20	135	10	44	10
805.6	184	20	135	10	44	10
806.6	184	20	135	10	44	10
807.6	184	20	135	10	44	10
808.6	184	20	136	10	44	10
809.7	184	20	136	10	44	10

810.7	184	20	136	10	44	10
811.7	184	20	136	10	44	10
812.7	184	20	136	10	44	10
813.7	184	20	137	10	45	10
814.7	184	20	137	10	45	10
815.7	184	20	137	10	45	10
816.7	184	20	138	10	45	10
817.7	184	20	138	10	45	10
818.7	184	20	138	10	45	10
819.7	184	20	138	10	45	10
820.7	184	20	139	10	45	10
821.7	184	20	139	10	45	10
822.7	184	20	139	10	45	10
823.7	184	20	139	10	45	10
824.7	184	20	140	10	45	10
825.7	184	20	140	10	45	10
826.7	184	20	140	10	46	10
827.7	184	20	140	10	46	10
828.7	184	20	141	10	46	10
829.7	184	20	141	10	46	10
830.7	184	20	141	10	46	10
831.7	184	20	141	10	46	10
832.7	184	20	142	10	46	10
833.7	184	20	142	10	46	10
834.7	184	20	143	10	46	10
835.7	184	20	143	10	46	10
836.7	184	20	143	10	47	10
837.7	184	20	143	10	47	10
838.7	184	20	144	10	47	10
839.7	184	20	144	10	47	10
840.7	184	20	144	10	47	10
841.7	184	20	144	10	47	10
842.7	184	20	144	10	47	10
843.7	184	20	145	10	47	10
844.7	184	20	145	10	47	10
845.7	184	20	145	10	47	10
846.7	184	20	146	10	48	10
847.7	184	20	146	10	48	10
848.7	184	20	146	10	48	10
849.7	184	20	146	10	48	10
850.7	184	20	147	10	48	10
851.7	184	20	147	10	48	10
852.7	184	20	148	10	48	10
853.7	184	20	148	10	48	10

854.7	184	20	148	10	48	10
855.7	184	20	148	10	48	10
856.7	184	20	149	10	48	10
857.7	184	20	149	10	48	10
858.7	184	20	141	10	46	10
859.7	184	20	141	10	46	10
860.7	184	20	150	10	49	10
861.7	184	20	150	10	49	10
862.7	184	20	150	10	49	10
863.7	184	20	150	10	49	10
864.7	184	20	151	10	49	10
865.7	184	20	151	10	49	10
866.7	184	20	152	10	50	10
867.7	184	20	152	10	50	10
868.7	184	20	152	10	50	10
869.7	184	20	152	10	50	10
870.7	184	20	153	10	50	10
871.7	184	20	153	10	50	10
872.7	184	20	153	10	50	10
873.7	184	20	153	10	50	10
874.7	184	20	154	10	50	10
875.7	184	20	154	10	50	10
876.7	184	20	154	10	50	10
877.7	184	20	154	10	50	10
878.7	184	20	154	10	50	10
879.7	184	20	154	10	50	10
880.7	184	20	154	10	50	10
